# Effectiveness of platelet-rich fibrin in third molar extractions: a randomized controlled split-mouth study

**DOI:** 10.1007/s00784-024-06002-9

**Published:** 2024-10-29

**Authors:** Katharina Zwittnig, Barbara Kirnbauer, Astrid Truschnegg, Norbert Jakse, Axel Wolf, Alwin Sokolowski, Irene Mischak, Michael Payer

**Affiliations:** 1 Division of Oral Surgery and Orthodontics, Department of Dental Medicine and Oral Health, Medical University of Graz, Graz, Austria; 2 Department of Otorhinolaryngology, Medical University of Graz, Graz, Austria; 3 Division of Restorative Dentistry, Periodontology and Prosthodontics, Department of Dental Medicine and Oral Health, Medical University of Graz, Graz, Austria

**Keywords:** Third molar surgery, Oral surgery, Platelet-rich-fibrin, Regenerative therapy

## Abstract

**Objectives:**

To date, studies have only investigated the use of platelet-rich fibrin (PRF) after removal of third molars from the mandible or maxilla. Removal of the upper and lower third molars is typically combined into one session per side; therefore, this study aimed to investigate influence on PRF.

**Materials and methods:**

This prospective, single-blinded, randomized controlled, clinical trial (split-mouth design) included 25 patients. After third molar removal, the test group’s sockets were treated with solid PRF clots, whereas the control group’s sockets were conventionally treated. The primary outcome was swelling, which was measured digitally and analogously. Secondary outcomes included trismus, pus, hematoma, and clinical attachment loss (CAL) of the second molars on days 1, 3, 7, and 14. Patient-centered outcome measures and the consumption of painkillers and antibiotics were recorded on days 0–7. The t-test for paired samples, Wilcoxon test, and Chi-Square test were used for statistical analyses.

**Results:**

Swelling was significantly lower on day 14 in the test group (*p < 0.05*). No statistically significant differences were observed in pain, trismus, and CAL. In the test group, the number of painkillers taken and the number of days of intake were significantly lower (*p < 0.05*).

**Conclusions:**

PRF caused a reduction of painkiller consumption and in the days painkillers were needed. PRF significantly considerably reduced swelling after 14 days. Owing to the lack of differences in other parameters, the integration of PRF application into routine wisdom tooth removal is critical.

**Clinical relevance:**

PRF affects the long-term outcomes of third molar removal by reducing swelling and reducing as well as shortening painkiller consumption.

**Trial registration:**

clinicaltrials.gov (NCT05089812).

## Introduction

Third molar removal is one of the most common treatments performed in oral surgery [1]. Clinical approaches, such as flap design and osteotomy, have been described since the early 20th century by Kells and Winter [2, 3]. Over the past few decades, surgical techniques have been considerably developed to prevent complications from extractions [1]. The most common complications are pain, trismus, edema, surgical site infections, and alveolar osteitis [4]. Several interventions have been described to limit the occurrence of these complications, including intake of painkillers and corticosteroids to minimize pain, swelling, and trismus. The application of cryotherapy and local chlorhexidine gel decreases the incidence of inflammatory side effects [4].

In recent decades, platelet-rich fibrin (PRF) has gained substantial interest in dentistry [5]. The release of growth factors from PRF can be detected in liquid and solid forms within the first few days after application [6–8]. Its easy and additive-free preparation has contributed to its increasing popularity in surgical procedures [6, 9]. Although the effect of PRF during mandibular third molar extraction has been studied, its impact on the aforementioned side effects after the extraction remains controversial [9, 10]. Furthermore, the application of PRF has not yet been investigated in patients who received simultaneous extraction of the upper and lower third molars. Therefore, current guidelines such as the German S3 guidelines (083–042) recommend the use of PRF for open alveolar healing and socket preservation. However, owing to insufficient data, a clear recommendation of using PRF to reduce postoperative pain cannot be made in the daily clinical routine [10, 11].

The null hypothesis of this study was that the application of PRF in third molar sockets did not improve postoperative outcomes compared with conventional methods.

A prospective randomized split-mouth design study was conducted, considering swelling as the primary outcome and signs of inflammation, such as the appearance of pus, hematoma, and wound dehiscence, as secondary outcomes. In addition, patients’ subjective perceptions were surveyed using questionnaires. This study aimed to explore a potential treatment option that leads to better clinical outcomes and fewer complications for patients than conventional treatment. The purpose of the trial was to compare the effort and benefits and to increase patients’ knowledge about the use of blood concentrates in dental medicine.

## Methods

### Study design

This prospective randomized clinical trial was a single-center, controlled study with a split-mouth design to determine if PRF influences the postoperative conditions of patients undergoing third molar extractions. This study was conducted at the Division of Oral Surgery and Orthodontics of the Department of Dental Medicine and Oral Health, Medical University of Graz, Austria. This study was conducted according to the Declaration of Helsinki and was approved by the Institutional Ethics Committee (protocol code: 33–451 ex 20/21; approval date: 08.19.2021).

### Patient recruitment, randomization, and blinding

This study included 25 healthy patients (American Society of Anesthesiologists classification, ASA1) aged 16 years or older with four noninfected, impacted, or partially impacted wisdom teeth. The participants were recruited from a routine outpatient clinic without any additional effort. The participants were nonsmokers or light smokers (less than 10 cigarettes a day) with no intake of anticoagulants. The position and level of impaction of the third molars were classified using the classifications proposed by Pell and Gregory [12] and Winter [3]. The level of surgical difficulty was similar on both sides for each patient to ensure comparability. All extracted wisdom teeth were free of cystic lesions and were not infected. Pregnant patients were excluded from this study. Furthermore, patients with immunosuppression or severe underlying diseases, such as diabetes mellitus, immunodeficiency, and advanced systemic disease, were excluded. Patients treated with therapies for diseases that affect bone or soft tissue metabolism (e.g., bisphosphonate therapy or localized radiotherapy) were also excluded. To ensure proper development of the wisdom teeth, patients 16 years of age or older were included. Preparation for the study, calibration meetings for all surgeons and investigators were held in order to standardize the surgical protocol as well as postsurgical assessments.

After providing informed consent at the first study visit (V1), each patient underwent two surgical procedures involving the extraction of the upper and lower third molars on either the left or right side. Randomization to test or control treatment was performed at the first surgical appointment using a web-based randomizer, which automatically allocated to the other group at the second surgery [13].

In the first surgery (V2), treatment of the surgical site after the extraction was conventionally performed using a gelatin sponge (Spongostan Dental, Johnson and Johnson, New Brunswick, New Jersey) to fill the defect (control group) or using a PRF clot to fill the sockets (test group) according to randomization. In the second surgery (V7), the other treatment method was used. At least 4 weeks were spent between the two treatments. Taking a sample of blood from the vein to create PRF clots was crucial. Blood was centrifuged at 1200 rpm for 8 min (177 g) [14, 15] within a centrifuge with a fixed angled rotor at a radius size of 110 mm [8]. To create solid PRF clots, 10-ml sterile glass tubes (A-PRF tubes Process for PRF™, Nice, France; Mectron, Cologne, Germany) were used [8]. To enable blinding of the participants, blood samples were also collected during surgery in the control group. Samples were collected to determine coagulation values and blood counts. Blood counts were obtained from the patients upon request. Because they were irrelevant to this study, no evaluations were conducted in this regard. Surgeons were not blinded as they had to applicate PRF or not during the surgery. The surgeons were informed on the day of surgery which group the procedure was assigned to. In total, patients underwent 11 visits (V1–V11). V1 was used to provide information, collect preoperative data, conduct a preliminary examination, and obtain informed consent. Panoramic X-rays were routinely taken for planning the surgical procedure (V1). In addition, the randomization procedure, which was performed without informing the patients to ensure blinding, determined the treatment allocation for each patient.

### Surgical procedures

At the second visit (V2), the surgical procedure on the first side was performed. As the experience of the surgeon has a decisive influence on the postoperative outcome of the patients [16], the operations were performed by two experienced oral surgeons with more than 15 years of professional experience. All patients underwent a preoperative disinfecting mouthwash rinse (Listerine cool mint, Johnson and Johnson, New Brunswick, New Jersey). On the day of the first and second surgeries, all patients orally received 40 mg of methylprednisolone (Urbason 40 mg, Sanofi-Aventis, Frankfurt am Main, Germany). Local anesthesia was applied to block Nervus (N.) buccalis, N. alveolaris inferior, and N. palatinus (Ultracain dental forte, articaine, and epinephrine 1:100 000, Normon S.A. Tres Cantos, Madrid, Spain). The upper wisdom tooth was exposed through an incision using the number 15 scalpel blade and removed using surgical elevators or forceps. The bone covering the buccal surface of the tooth was carefully removed using osteotomy burs with a hand piece and surgical engine (W&H^®^ Implantmed, W&H Dentalwerk, Bürmoos GmbH, Austria). An envelope flap was used to expose the lower third molar. After raising the mucoperiosteal flap, the tooth was bared and extracted using elevators or forceps. If required, the bone was removed using a round drill. To reduce surgical trauma, if necessary, the lower wisdom tooth was carefully split and retrieved in separate components. After removing the tooth follicle and rinsing with saline solution, the wound was closed with nonresorbable stitches (5.0 Dafilon, B.Braun). Dexibuprofen (400 mg Seractil forte film-coated tablets, Gebro Pharma, Fieberbrunn, Austria) up to three times a day were prescribed for on demand intake. Each surgery was followed by four control examinations (V3–V6 and V8–V11) on the 1st, 3rd, 7th, and 14th postoperative days. On the 7th postoperative day, the nonresorbable sutures (5.0 Dafilon, B.Braun, Tuttlingen, Germany) were removed. Every postoperative visit included analog and digital measurements of the swelling and evaluation of inflammation signs [17]. Clinical assessment included evaluation of swelling, probing depth of the second molars, interincisal distance to assess trismus, and examination to evaluate secretion, dehiscence, or hematoma. Probing depth distal to the second molars, which were measured with a periodontal probe and compared by calculating the difference between preoperative and postoperative measurements in the test versus control groups. The greater the difference, the worse the outcome. Trismus was evaluated by measuring the interincisal distance using a measurement tape. Swelling was evaluated from the summation of the following measurements using a measurement tape: outer eye corner to mandibular jaw, tragus to mouth corner, and tragus to the foremost point of the chin symphysis [17]. Digital edema was measured by superimposing pre- and postoperative 3D face scans (Planmeca ProMax 3D Max, Planmeca Oy, Helsinki, Finnland). Volume differences in milliliters (ml) were determined using the coDiagnosticX software (Dental Wings GmbH, Chemnitz, Germany) by manually superimposing the sections in a voxel size of 400 μm [17].

Patient-reported outcome measures were evaluated through questionnaires. Bleeding, swelling, and pain were rated by each patient daily during the 1st postoperative week on a visual analog scale (VAS) from 1 to 10. Patients also had to indicate which medications they were taking and in what quantities. On day 14, another questionnaire with a numerical scale from 1 to 10 was to be completed to evaluate pain, swelling, bleeding, and patient satisfaction.

## Statistical analysis of the outcome measures

After a comprehensive literature review and analysis of the study protocols, a power analysis was conducted using the data of postoperative swelling from Gupta et al. (2021) [18]. A sample size of 18 had 80% power to detect a difference in means of 0.27 mm (3.73 mm [mean control] and 2.46 mm [mean PRF]), assuming a standard deviation of differences of 0.38 mm, and a paired t-test with a 5% two-sided significance level. Owing to limited data and experience with dropout rates for PRF split-mouth studies, a sample size of 25 patients with 50 interventions was planned. Statistical analyses were performed using the Statistical Package for the Social Sciences software (IBM SPSS statistics 29.0, IBM Corporation, New York). A t-test for paired samples was applied to analyze digitally and analogously measured edema, intake of painkillers, bleeding, patient satisfaction, and pain evaluation using numeric scales. Furthermore, this statistical test was applied to analyze the trismus and CAL of the second molar. The presence or absence of wound dehiscence was evaluated using the Chi-Square test. The incidence of hematoma, wound secretions, and pus was statistically assessed using the Wilcoxon test. Linear regression analysis (effect size and 95% confidence interval) was performed for the primary outcome (analog and digital swelling). For analog swelling, the regression analysis was conducted comparing the entire course with the change over time for the factors gender and solid PRF application. For digital swelling measurements, the linear regression was analyzed comparing swelling at for different timepoints (difference between preoperative measurement and measurement at day 1,3,7, or 14 post surgery). A general linear regression model with repeated measurements was used to test for differences in probing depths of the second molar between upper and lower jaw.

## Results

**Fig. 1:**
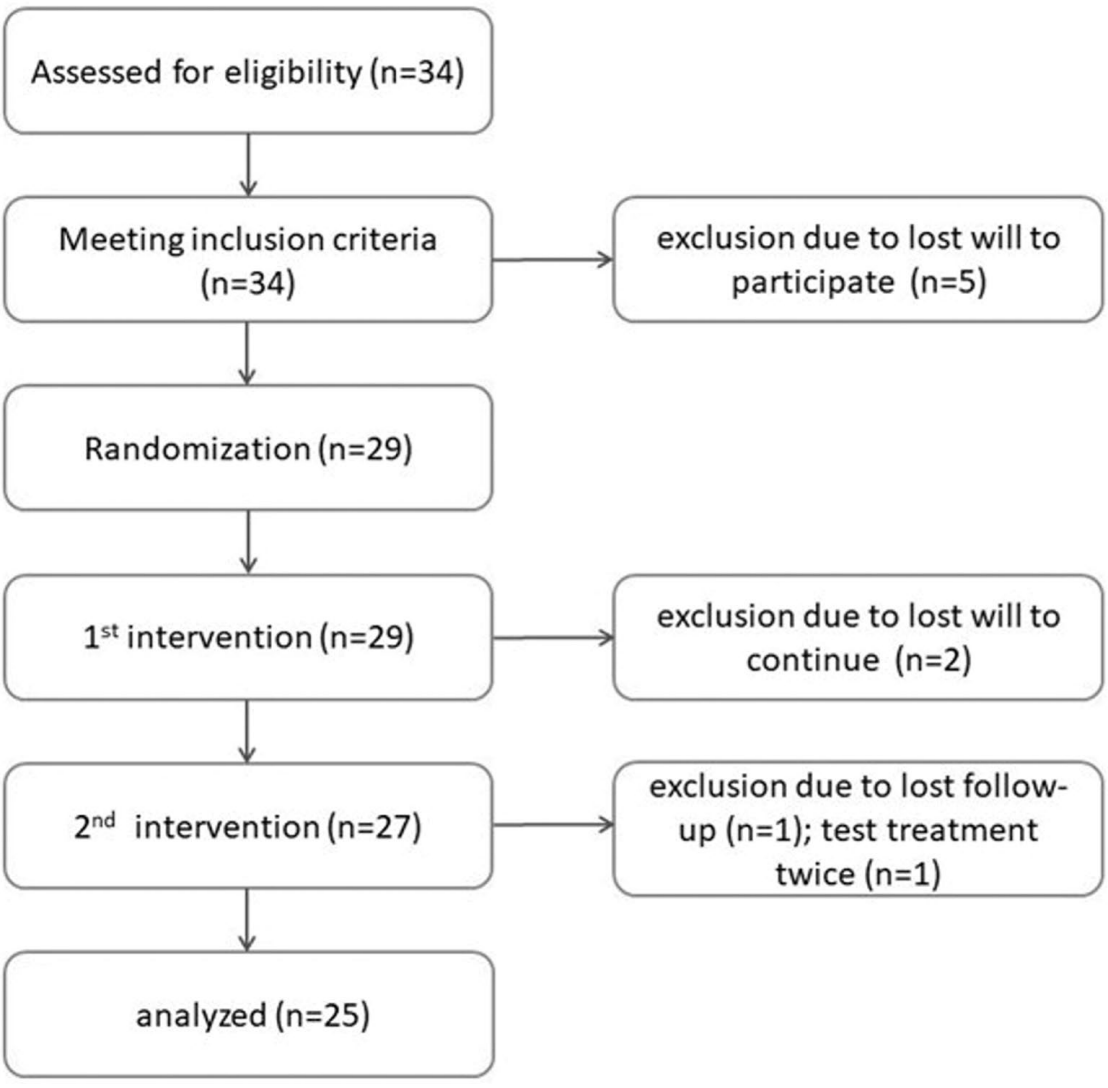
CONSORT flow chart showing information about enrollment (34 patients were assessed for eligibility and met the inclusion criteria, 5 were excluded owing to loss of interest), randomization (29 patients were randomized), allocation and interventions (as 2 participants lost the will to continue, 27 patients underwent the second surgery), and analysis (owing to the loss of follow-up [*n* = 1] and undergoing test treatment twice [*n* = 1]; 25 patients were analyzed)

A total of 34 patients were recruited for this study. Of these, seven patients dropped out owing to withdrawal consent, one was treated with PRF twice, and one was excluded owing to lost follow-up. Only patients treated according to the study design were included in the analysis (Fig. 1).

In total, 25 patient results were analyzed in this split-mouth study. The study population comprised 76% female (*n* = 19) and 24% male (*n* = 6) healthy participants. One patient included was light smoker (less than 10 cigarettes per day). The total duration of this study was 33 months. The number of extracted wisdom teeth was 100, fifty teeth in each group. The flap creation was performed if wisdom teeth were fully or partly impacted and could not be extracted otherwise (this was the case in all 50 lower wisdom teeth and 47 out of 50 upper wisdom teeth, no flap was necessary for two upper wisdom teeth in the test group and one in the control group).

### Primary outcome variable

**Fig. 2:**
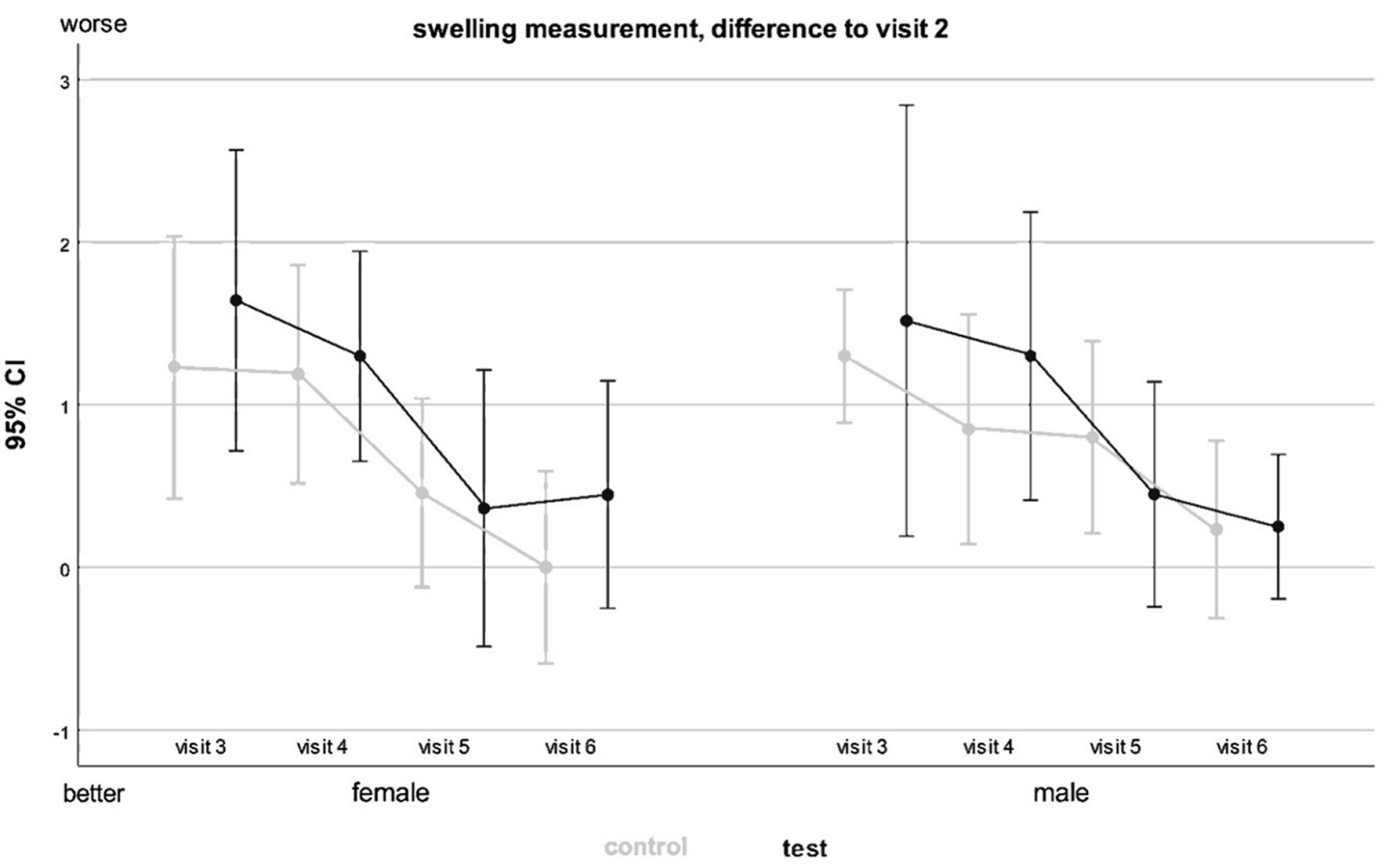
Influence of gender on analogue swelling measurement (grey: conventional treatment, black: PRF treatment)

To evaluate the analog swelling measurements using a measuring tape, the three measured lengths were added together and the sum was compared with the value of the preoperative visit [17]. The lower the difference, the smaller the edema. At all time points, no statistically significant differences were detected (*p > 0.05*) (Table 1). Linear regression did not detect significant differences in analog swelling measurement regarding solid PRF treatment or gender (*p* < 0.05) (Fig. 2).

**Table 1:** Analog swelling measurements

Visit	Group	*N*	Min	Max	Mean	SD	*p*-value*
V03–V02	Control	23	−1	5,1	1,25	1,35	*0*,*373*
	Test	20	−0,7	6,3	1,61	1,48	
V04–V02	Control	21	−1,6	4,1	1,12	1,19	*0*,*947*
	Test	22	−0,2	4,6	1,30	1,10	
V05–V02	Control	25	−1,9	3,4	0,54	1,08	*0*,*568*
	Test	25	−2,6	5,9	0,38	1,56	
V06–V02	Control	23	−1,9	1,7	0,06	1,02	*0*,*266*
	Test	23	−1	4,7	0,40	1,18	

Summary of analog swelling measurements (using a measurement tape: outer eye corner to mandibular jaw, tragus to mouth corner and tragus to the foremost point of the chin symphysis) compared with preoperative measurements on days 1 (V01–V03), 3 (V01–V04), 7 (V01–V05), and 14 (V01–V06). Mean values (mean), standard deviations (SD), significance level (p), number of patients (N), control (conventional treatment), test (PRF application) (*t-test for paired samples)

Swelling measured with the superimposition of 3D face scans showed a significantly lower volume difference in ml at the last visit (day 14) than in the control group (*p < 0.05*) (Table 2). During the other visits, slightly lower levels of swelling were observed in the control group; however, these results were not significant (*p > 0.05*). Linear regression did not detect significant differences in digital swelling measurement regarding solid PRF treatment or gender for any timepoint (*p* > 0.05).

**Table 2:** Swelling using 3D meassurement

Visit	Group	*N*	Min	Max	Mean	SD	*p*-value*
V01–V03	Control	22	3,10	47,52	17,87	12,81	*0*,*382*
	Test	18	4,78	69,25	18,71	15,77	
V01–V04	Control	18	3,99	48,67	17,04	11,35	*0*,*784*
	Test	20	4,69	55,07	18,17	13,03	
V01–V05	Control	23	3,07	22,90	9,27	5,32	*0*,*948*
	Test	24	2,35	33,71	10,40	8,31	
V01–V06	Control	19	0,53	20,53	8,62	4,09	***0***,***049***
	Test	22	2,60	27,95	7,11	5,49	

Swelling 3D scan comparing preoperative face scan with scans on days 1 (V01–V03), 3 (V01–V04), 7 (V01–V05), and 14 (V01–V06) (difference of volume in ml). Mean values (mean), standard deviations (SD), significance level (p), number of patients (N), control (conventional treatment), test (PRF application) (t-test for paired samples)

## Secondary outcome variables

**Fig. 3:**
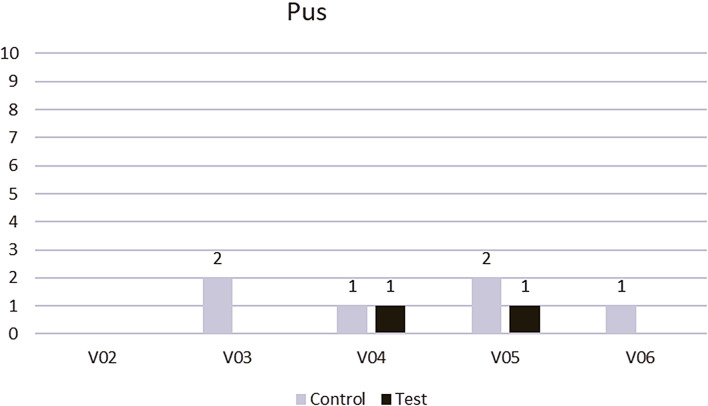
Pus was recognized more frequently in the control group (conventional treatment; gray) than the test group (PRF application; black) (Wilcoxon test)

Pus was recorded more frequently in the control group; however, the differences were not significant owing to the rarity of the event (Table 3; Fig. 3).

**Table 3:** Appearance of pus

Pus	*N*	Mean	Median	*p*-value*
Control	6	0,24	0	*0*,*157*
Test	2	0,08	0	

Appearance of pus shows no significant differences. Mean values (mean), significance level p, number of patients (N), control (conventional treatment), test (PRF application) (*Wilcoxon test)

**Fig. 4:**
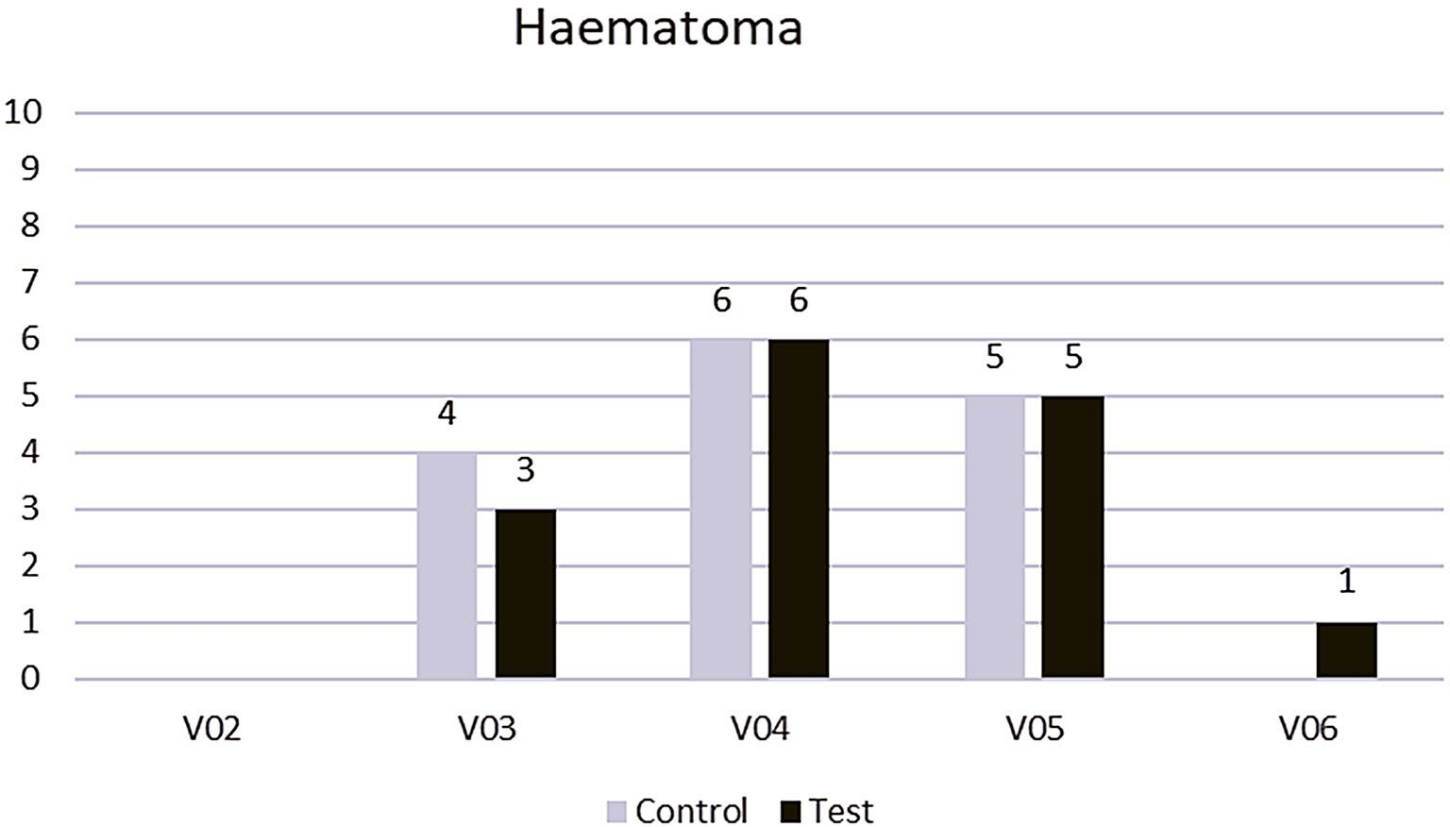
Appearance of the hematoma in the control group (conventional treatment; gray) vs. test group (PRF application; black)

Hematoma occurred more frequently in the control group on days 1 (V03) and 3 (V04); however, the difference was not significant (Fig. 4; Table 4).

**Table 4:** Appearance of hematoma

Hematoma	Control		Test	
	N	Yes	N	Yes
Day 0	25	0	25	0
Day 1	23	4	20	3
Day 3	21	6	22	6
Day 7	25	5	25	5
Day 14	23	0	23	1
Hematoma	Sum	Mean	Median	*p*-value*
control	15	0,60	0	*0*,*904*
test	15	0,60	0	

Number of patients who attended this visit (N), hematoma occurred (Yes), control (conventional treatment), test (PRF application) (*p-value was determined using the Wilcoxon test)

Furthermore, no significant differences were observed in secretion between the two groups (Table 5).

**Table 5:** Secretion of none, clear or turbid liquid

Visit	Group	Turbid	Clear	Non	*p*-value*
preOP			1	24	
V03	Control	3	6	14	*0*,*782*
	Test	2	6	12	
V04	Control	2	1	18	*0*,*482*
	Test	3	3	16	
V05	Control	4	1	20	*0*,*435*
	Test	2	2	21	
V06	Control	2	1	20	*0*,*194*
	Test		1	22	

Significance level (p) (*Wilcoxon test)

## Significance level (p) (*Wilcoxon test)

On the 1st and 3rd postoperative days, the test group had higher values for mouth opening. On the 3rd postoperative day, the difference was statistically significant. This indicates that mouth opening was considerably less restricted in the test group on day 3 than in the control group (Table 6).

**Table 6:** Evaluation of trismus

Visit	Group	*N*	Min	Max	Mean	SD	*p*-value*
preOP		25	4	7	5,02	0,76	
Day 1	Control	23	2,2	5,7	3,64	0,96	*0.200.*
	Test	20	2	5	3,68	0,88	
Day 3	Control	21	2,7	5	3,74	0,69	***0.021***,
	Test	22	3	6,6	4,33	1,03	
Day 7	Control	25	3	7,1	4,38	1,04	*0.359.*
	Test	25	2,2	6,5	4,43	1,01	
Day 14	Control	23	3,2	7	4,74	0,92	*0*,*116*
	Test	23	3,4	6,5	4,83	0,88	

Evaluation of trismus within the measurement of distance between upper and lower incisive using a measuring tape. Mean values (mean), standard deviations (SD), significance level (p), number of patients (N), control (conventional treatment), test (PRF application) (*t-test fot paired sample)

**Fig. 5:**
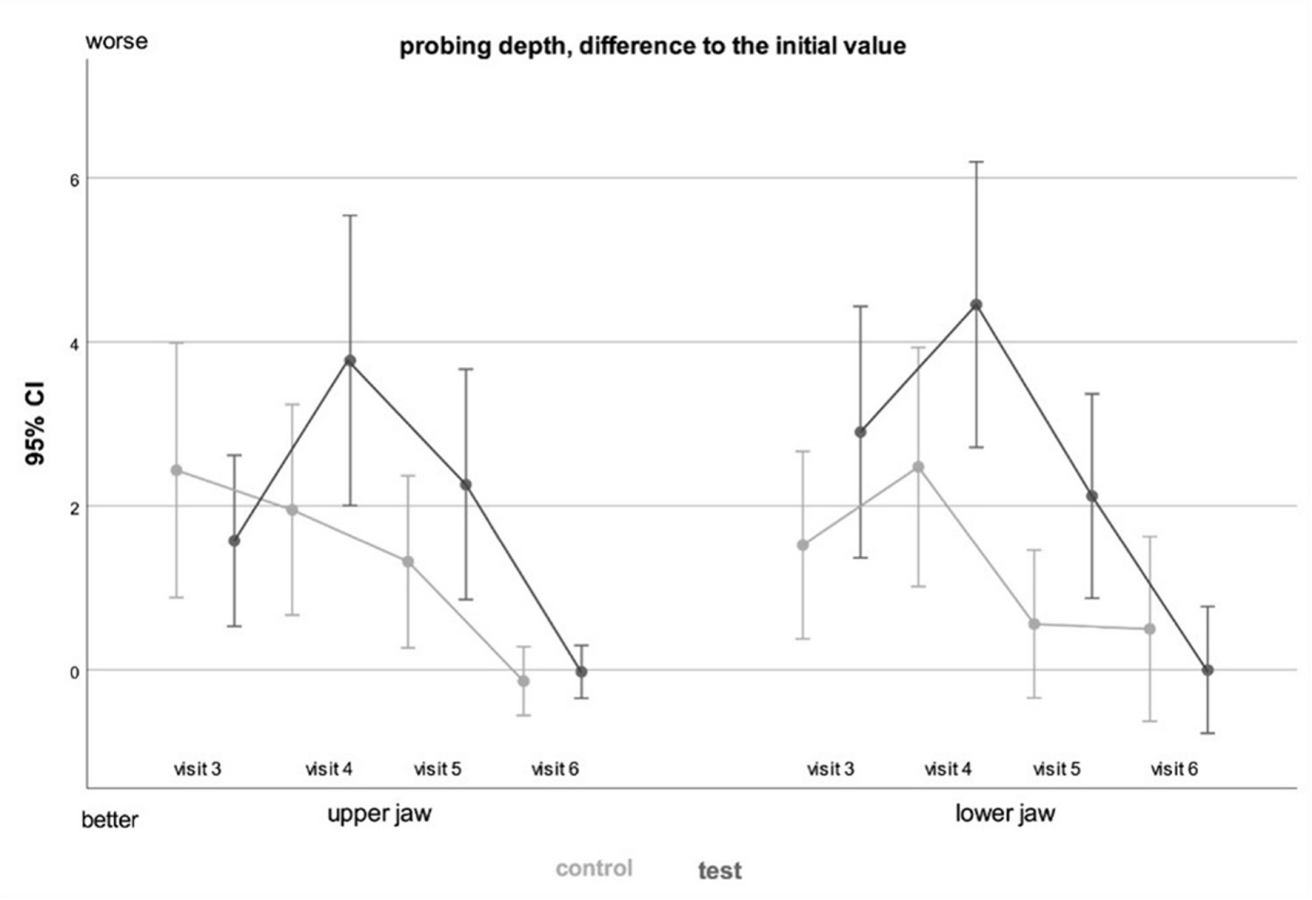
General linear model with repeated measurements comparing effect of PRF in upper vs. lower jaw (grey: control group, black: test group)

Probing depths were measured distal of the second molar. This outcome variable has a very high dispersion. Therefore, the statistical test is not highly conclusive. On day 7, the probing depths of the lower third molars were significantly higher in the test group (*p < 0.05*) (Table 7). There was no statistically significant difference in the effect of PRF neither in upper nor the lower jaw (Fig. 5).

**Table 7:** Probing depths of the second molars

Visit	Group	*N*	Min	Max	Mean	SD	*p*-value*
Day 1 17/27	Control	23	0	10,5	2,41	3,49	*0*,*196*
	Test	20	−0,5	6,5	1,60	2,20	
Day 1 37/47	Control	23	−2	8,5	1,70	2,28	*0*,*274*
	Test	20	−3	9	2,75	3,44	
Day 3 17/27	Control	21	−1	9,5	1,98	2,69	*0*,*225*
	Test	22	0	12,5	3,82	3,94	
Day 3 37/47	Control	21	−2,5	9	2,45	2,98	*0*,*054*
	Test	22	−0,5	11,5	4,34	3,84	
Day 7 17/27	Control	25	−1	9,5	1,30	2,39	*0*,*279*
	Test	25	−1	11	2,30	3,45	
Day 7 37/47	Control	25	−2,5	7	0,66	1,81	***0***,***046***
	Test	25	−4	9	2,02	3,15	
Day 14 17/27	Control	22	−1,5	1,5	−0,16	0,73	*0*,*748*
	Test	23	−1,5	1,5	0,02	0,76	
Day 14 37/47	Control	22	−3	6,5	0,61	2,20	*0*,*138*
	Test	23	−5	6	−0,13	2,09	

Probing depths of the second molars (right side: 17/47 and left side: 28/38) preoperatively vs. postoperatively on days 1, 3, 7, and 14. Mean values (mean), standard deviations (SD), significance level (p), number of patients (N), control (conventional treatment), test (PRF application) (*t-test for paired samples)

Soft tissue healing was determined by whether the dehiscence was detectable. Statistical analyses using the Chi-squared test revealed no significant differences between the two groups (*p > 0.05*) (Table 8).

**Table 8:** Soft tissue healing

	Control			Test			*p*-value*
	n	Dehicent/not completely covered	Non-dehiscent/ completely covered	n	Dehicent/not completely covered	Non-dehiscent/ completely covered	
V03 18/28	23	3	20	20	2	18	*0*,*216*
V04 18/28	21	2	19	22	3	19	*0*,*331*
V05 18/28	25	5	20	25	5	20	*0*,*252*
V06 18/28	23	3	20	23	3	20	*0*,*386*
V03 28/38	23	4	19	20	5	15	*0*,*650*
V04 28/38	21	10	11	22	9	13	*0*,*419*
V05 28/38	25	17	8	25	17	8	*0*,*513*
V06 28/38	23	18	5	23	15	8	*0*,*656*

Soft tissue healing determined whether the wound is dehiscent or fully closed and evaluated postoperatively from day 0 (day of surgery) until day 14. Mean values (mean), standard deviations (SD), significance level (p), control (conventional treatment), test (PRF application) (*Chi-Square test)

### Patient-centered outcome variables

For pain relief, patients took significantly more pills in the control group (*p < 0.05*). Furthermore, *medication* was taken on significantly more days in the control group (*p < 0.05*) (Table 9). There were no known allergies against painkillers. Seven patients reported deviations from the painkiller prescription (dexibuprofen): three patients reported the additional intake of paracetamol, metamizole, ibuprofen, mefenamic acid or combinations of those after both, test and control treatment. Another patient used dexketoprofen instead of dexibuprofen after test treatment, and ibuprofen instead of dexibuprofen after control treatment; another patient reported the additional intake of paracetamol after control treatment; two patients reported the additional intake of ibuprofen and replaced dexibuprofen with ibuprofen for several timepoints after control treatment; finally, one patient additionally used mefenamic acid after test treatment.

In the test group, one patient was treated with antibiotics owing to infection, and in the control group, three participants were administered antibiotic therapy. Notably, one patient in the test and control group required antibiotics.

**Table 9:** Painkiller intake

Medication	Group		Mean	SD	*p*-value*
Quantity of Painkillers	Control		15,8	7,5	***0***,***007***
	Test		12,5	5,4	
Days of Intake	Control		6,6	2,1	***0***,***044***
	Test		5,8	1,9	

Quantity and days of painkiller intake evaluated postoperatively from day 0 (day of surgery) until day 14. Mean values (mean), standard deviations (SD), significance level (p), control (conventional treatment), test (PRF application) (*t-test for paired samples)

**Fig. 6:**
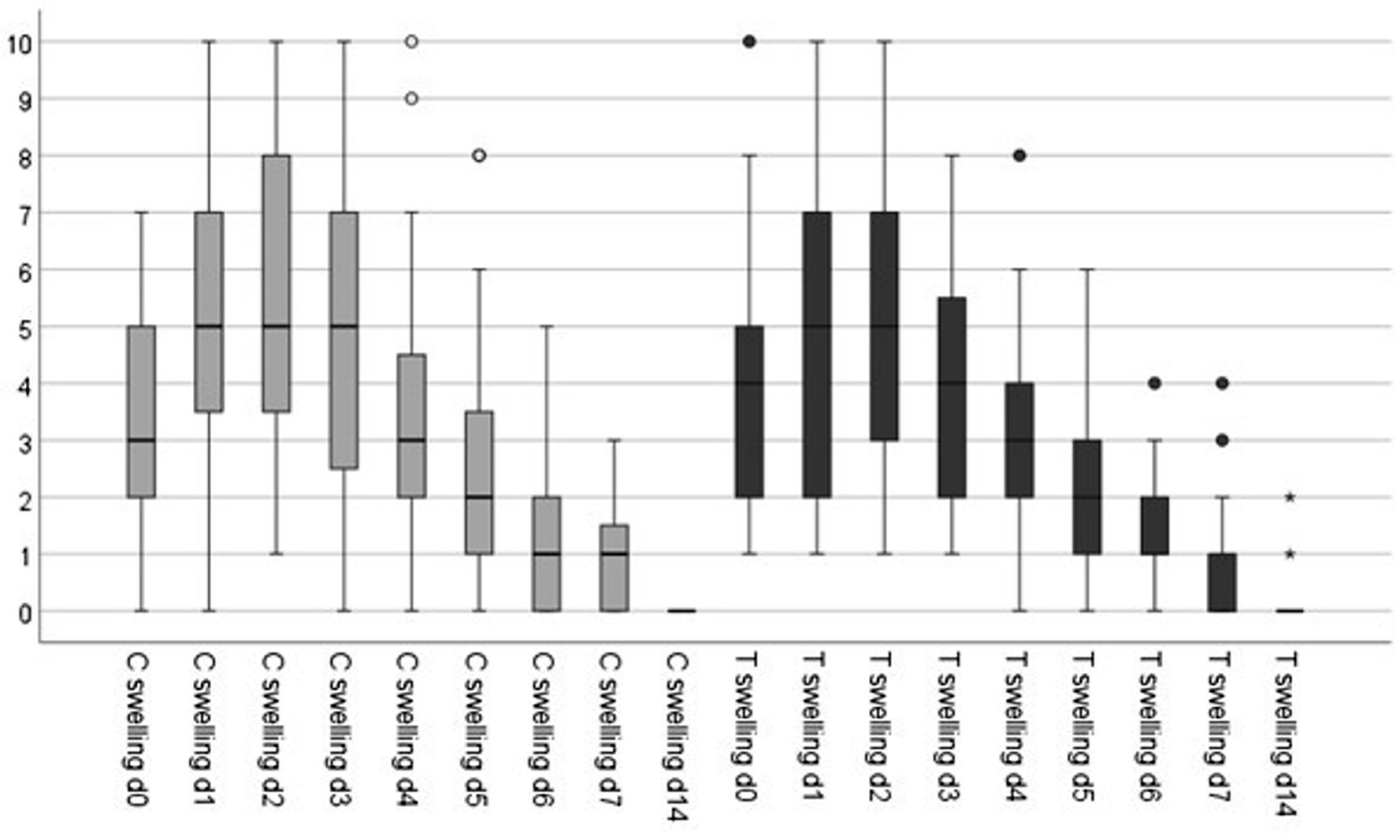
Subjective swelling evaluated using a numeric rating scale comparing the control group (C; gray) with the test group (T; black) postoperatively from day 0 (day of surgery) until day 14. (t-test for paired samples)

**Fig. 7:**
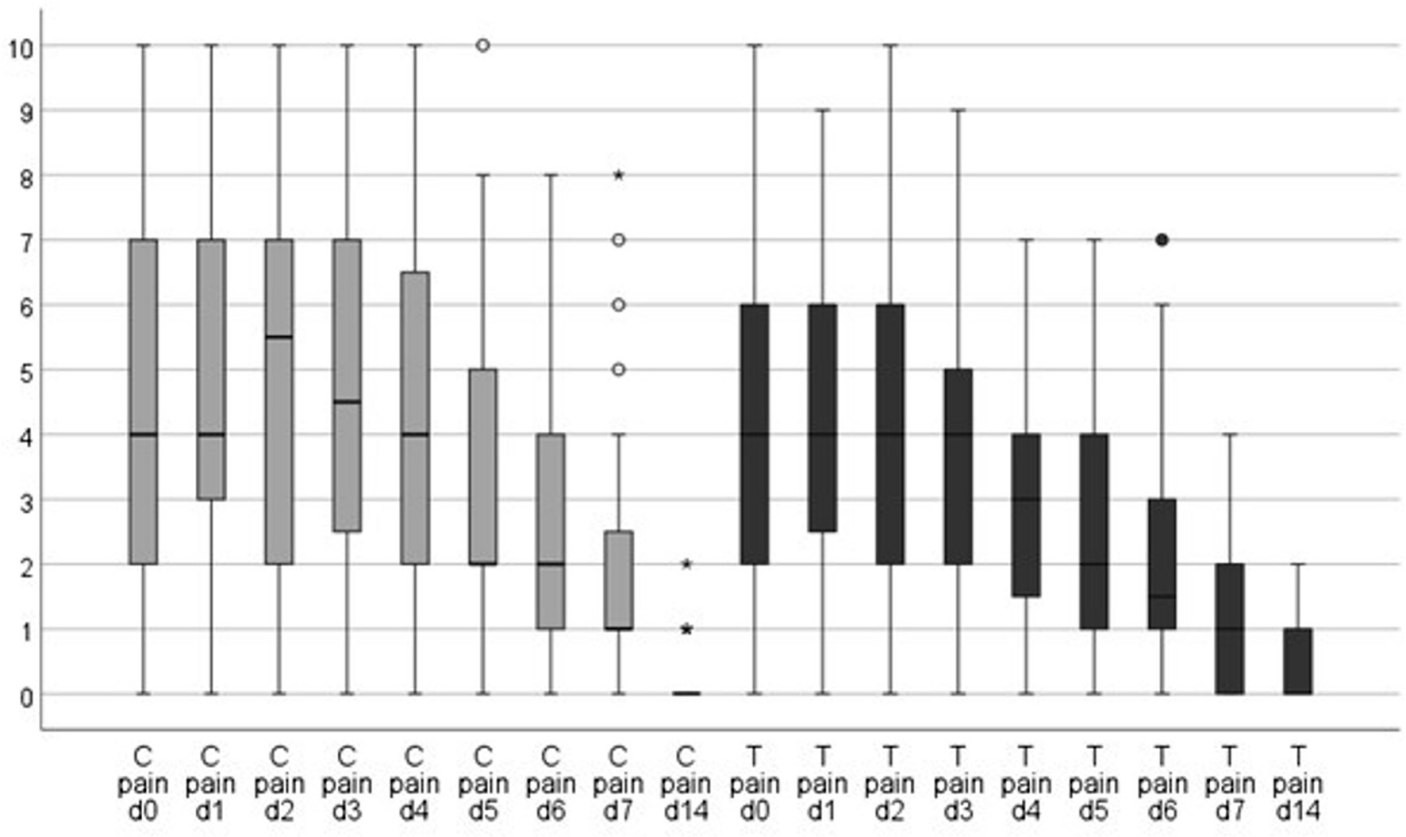
VAS of pain evaluated in the 1st week and on day 14: control group (C; gray) vs. test group (T; black). Lower pain levels were observed in the test group (PRF) at all time points

The perceptions of pain, swelling, and bleeding was assessed using numeric rating scales. The subjective perception of bleeding according to the questionnaire tended to be higher in the test group. This result was significant on day 1 (*p < 0.05*) (Table 10). A tendency was observed toward higher subjective swelling in the control group on all days surveyed (Table 11; Fig. 6). However, the values were not significant (*p > 0.05*). The pain scale had slightly lower values in the test group. These results were marginally nonsignificant (*p > 0.05*) (Table 12; Fig. 7).

**Table 10:** Evaluation of bleeding

	Control				Test				
	Min	Max	Mean	SD	Min	Max	Mean	SD	*p*-value*
Day 0	0	7	3,48	2,16	0	10	4,04	2,69	*0*,*248*
Day 1	0	4	1,20	1,29	0	7	2,20	2,16	***0***,***038***
Day 2	0	3	0,84	0,90	0	9	1,36	2,00	*0*,*211*
Day 3	0	4	0,72	1,06	0	3	0,60	0,76	*0*,*612*
Day 4	0	3	0,40	0,76	0	4	0,64	1,32	*0*,*256*
Day 5	0	1	0,12	0,33	0	3	0,28	0,68	*0*,*327*
Day 6	0	1	0,04	0,20	0	2	0,08	0,40	*0*,*664*
Day 7	0	1	0,04	0,20	0	0	0,00	0,00	*0*,*327*
Day 14	0	0	0,00	0,00	0	1	0,05	0,21	*0*,*330*

Bleeding evaluated using a numeric rating scale postoperatively from day 0 (day of surgery) until day 14. Mean values (mean), standard deviations (SD), significance level (p), control (conventional treatment), test (PRF application) (t-test for paired samples)

**Table 11:** Evaluation of subjective swelling

	Control				Test				
	Min	Max	Mean	SD	Min	Max	Mean	SD	*p*-value*
Day 0	0	7	3,24	2,07	1	10	3,96	2,35	*0*,*262*
Day 1	1	10	5,08	2,52	1	10	4,96	2,94	*0*,*837*
Day 2	1	10	5,56	2,95	1	10	5,36	2,58	*0*,*709*
Day 3	0	10	4,92	2,97	1	8	4,16	2,21	*0*,*210*
Day 4	0	10	3,56	2,55	0	8	3,08	1,85	*0*,*344*
Day 5	0	8	2,68	2,38	0	6	1,88	1,36	*0*,*099*
Day 6	0	5	1,64	1,47	0	4	1,24	1,01	*0*,*260*
Day 7	0	3	1,00	1,04	0	4	0,64	0,95	*0*,*131*
Day 14	0	0	0,00	0,00	0	2	0,14	0,47	*0*,*186*

Subjective swelling evaluated using a numeric rating scale postoperatively from day 0 (day of surgery) until day 14. Mean values (mean), standard deviations (SD), significance level (p), control (conventional treatment), test (PRF application) (t-test for paired samples)

**Table 12:** Evaluation of pain

	Control				Test				
	Min	Max	Mean	SD	Min	Max	Mean	SD	*p*-value*
Day 0	0	10	4,80	3,10	0	10	4,71	2,82	*0*,*784*
Day 1	0	10	4,68	2,80	0	9	4,20	2,16	*0*,*407*
Day 2	0	10	5,12	2,88	0	10	4,21	2,59	*0*,*179*
Day 3	0	10	5,00	2,87	0	9	3,84	2,39	*0*,*095*
Day 4	0	10	4,12	2,68	0	7	3,04	2,07	*0*,*058*
Day 5	0	10	3,36	2,41	0	7	2,44	2,18	*0*,*115*
Day 6	0	7	2,56	1,98	0	7	1,92	1,89	*0*,*155*
Day 7	0	7	1,96	1,79	0	4	1,24	1,23	*0*,*059*
Day 14	0	2	0,30	0,56	0	2	0,36	0,66	*0*,*453*

VAS evaluating pain evaluated in the 1st week and on day 14 postoperatively from day 0 (day of surgery) until day 14. Mean values (mean), standard deviations (SD), significance level (p), control (conventional treatment), test (PRF application) (t-test for paired samples)

No significant differences were observed in patient satisfaction after 14 days (Table 13).

**Table 13:** Patient satisfaction after 14 days

	Control				Test				
	Min	Max	Mean	SD	Min	Max	Mean	SD	*p*-value*
Day 14	5	10	9,00	1,48	5	10	9,14	1,42	*0*,*878*

Mean values (mean), standard deviations (SD), significance level (p), control (conventional treatment), test (PRF application) (*t-test for paired samples)

## Discussion

The following null hypotheses can be discussed in this study: PRF application does not reduce swelling in the first two weeks after third molar removal. This hypothesis was contradicted, as significantly less swelling was observed on day 14 in the PRF group (*p* < 0.05) measured three-dimensional. The null hypothesis, that PRF application has no influence on the consumption of painkillers was not confirmed, as it was significantly lower in the test group than in the control group (*p* < 0.05). Additionally, the null hypothesis, that PRF has no impact on trismus could be refuted, as mouth opening was significantly less restricted in the test group on day three (*p* < 0.05). These null hypotheses were confirmed: PRF has no significant impact on pain perceived by patients, soft tissue healing, hematoma, appearance of pus and distal probing depths of the second molars.

As of this date, a review of the literature using PubMed reveals that 22 randomized controlled clinical trials investigating third molar extractions and the use of PRF have been published. Of these, 16 study groups applied a split-mouth design [19–34]. None of the patients underwent the upper and lower third molar extractions, and in only one study patients underwent the upper wisdom tooth extraction [22]. The combination of upper and lower wisdom tooth removal in one operation has the advantage that the patient only needs two surgeries. However, if only the lower wisdom teeth are removed, several surgeries are required to extract all four wisdom teeth. Thus, from a practical and patient-oriented perspective, the approach of this study appears favorable.

The study by Zahid et al. had 10 participants. Zahid et al. reported the lowest number of cases among the studies mentioned [28]. The highest number of cases included in the split-mouth-designed studies that evaluated edema was 70 participants [21]. Praganta et al. reported no statistically significant differences between the test and control groups regarding pain or edema [21]. This result is remarkable owing to the high number of cases and is consistent with the findings of this study. Two additional research groups evaluated swelling by 3D examination [24, 26]. Similar to this study, these two studies did not report any statistically significant differences in edema during the first postoperative week [24, 26]. By contrast, 10 clinical trials using an analog measurement of swelling found statistically significant lower values in the PRF group [20, 25, 28, 29, 33–38].

Analgesic consumption was analyzed in three previous studies [34, 35, 39]. Of these, one study reported statistically significantly lower values of analgesic consumption comparable to the findings in the present [34].

None of the 22 studies investigated the need for additional antibiotic intake. Herein, three patients were required to take antibiotics in the control group and one patient in the test group.

In seven studies, the periodontal tissue of the second molars was monitored using CAL and the periodontal probing depth (PPD) [19, 23, 28, 30, 32, 36, 38]. Two of them, Kumar et al. and Gasparro et al., reported considerably shallower probing depths of adjacent molars in the PRF group [19, 38]. Gasparro et al. evaluated three specimens recorded at three places (distal, disto-lingual, and disto-buccal). Herein, the probing depth at one point at the distal end of the second molar was measured. As in the trial of Gasparro et al., a manual periodontal probe was applied with about 0.2–20 g of probing pressure [19]. No statistically significant differences were observed in the probing depths of the second molars in this study.

Concerning postoperative pain, Daugela et al. reported significantly better results in the first week after surgery (*p < 0.05*) [20, 40]. Similar to this study, Praganta et al. found no statistically significant differences in pain during the first week [21]. Pain was evaluated using the VAS. In total, 8 out of 17 studies have shown considerably lower pain in the PRF group [20, 28–30, 33, 37, 38, 41]. In this investigation, pain values were collected in the first week and on day 14. These values were lower in the test group at all time points, and this result was not significant (Fig. 2).

Trismus was assessed using the interincisal distance between the upper and lower jaw in eight other studies. However, considerably lower values were observed in the PRF group in only three patients [34, 38, 41]. One of the studies did not have a split-mouth design but had two parallel groups [41]. At all time points, mouth opening was better in the test group. However, the difference was too small to be considered statistically significant.

The experience of surgeons has an impact on the patients’ outcomes after surgeries [16]. Therefore, herein, every extraction was performed by two doctors specialized in oral surgery and working in this field for more than 15 years to minimize bias.

The number of studies on PRF and its use in the third molar extractions shows a high interest in this topic. The data on the PRF application are still heterogeneous and difficult to compare owing to differences in study protocols. Therefore, so far in clinical trials, the lower wisdom teeth have been mainly examined [9, 10, 40, 42, 43]. Although, at present, in general, the upper and lower wisdom teeth are usually simultaneously removed on each side during one surgical intervention [17]. Given the disputed results and the few substantial differences in the results, recommending PRF as a daily routine for wisdom tooth surgery is not justifiable. However, some positive aspects of the PRF application can be identified. Nevertheless, the effort and time costs of preparation must be weighed against the rather small benefits. The reduced intake of painkillers speaks in favor of the method. This study has certain limitations. The sample size of our study was small. Moreover, owing to the wide variety of PRF manufacturing processes and protocols, the comparability between the studies is not directly feasible. An agreement on a protocol or further studies with the same manufacturing protocol would be important to substantiate the results of this study. Furthermore, the dropout rate in the current study was high (nine exclusions). According to the sample size calculation, the suggested number of participants could be analyzed to obtain reliable results. However, more studies with larger sample sizes are necessary to validate the existing results.

## Conclusion

This study showed consistently positive effects of PRF on postoperative outcomes. In particular, the reduction in the need for painkillers should be emphasized. Swelling was considerably lower in the test group on day 14. However, considering that substantially better results were observed for only a few clinical outcomes, PRF cannot be recommended as a standard procedure for wisdom tooth removal. The time required to produce the blood concentrate was not associated with weak positive effects. However, its application in selected cases such as patients with a high perception of pain could be reasonable. Further studies are required to make clear recommendations for or against this treatment.

## Data Availability

No datasets were generated or analysed during the current study.
